# High CENPM mRNA expression and its prognostic significance in hepatocellular carcinoma: a study based on data mining

**DOI:** 10.1186/s12935-020-01499-y

**Published:** 2020-08-26

**Authors:** Zeng-hong Wu, Dong-liang Yang

**Affiliations:** Department of Infectious Diseases, Union Hospital, Tongji Medical College, Huazhong University of Science and Technology, Wuhan, 430022 China

**Keywords:** Hepatocellular carcinoma, Centromere protein M, Data mining, Prognosis

## Abstract

**Background:**

Hepatocellular carcinoma (HCC) is a high mortality disease, the fifth most general cancer worldwide, and the second leading to cancer-related deaths, with more than 500,000 new patients diagnosed each year. First, the high expression of centromere M (*CENPM)* in mammary gland tissue of b-catenin transformed mice was identified.

**Materials and methods:**

In our study, we evaluated the expression of *CENPM* in hepatocellular carcinoma based on data obtained from an online database. Multivariate analysis showed that the expression of *CENPM* and M classification was an independent prognostic factor for patients with hepatocellular carcinoma.

**Results:**

Survival analysis showed that patients with high *CENPM* had a worse prognosis than patients with low *CENPM* (*P* < 0.01). A multivariate Cox regression hazard model showed that B cells, CD8+ T cells, macrophages, and dendritic cells infiltrated by immune cells were statistically significant in liver cancer (*P* < 0.05). Using the network, the 50 most frequently changed neighbor genes of CENPM were shown, and the most common change was *RAD21* (18.3%).

**Conclusion:**

Our study found that the expression of *CENPM* was significantly increased in patients with hepatocellular carcinoma, and it was related to a variety of clinical characteristics, its correlation with the level of immune infiltration and poor prognosis, so *CENPM* can be used as a useful prognosis for patients’ markers and HCC**.**

## Background

Hepatocellular carcinoma (HCC), a high mortality disease which is the fifth most general cancer in the world and the second most common lead to cancer-related deaths, with over 500,000 new patients diagnosed each year [1, 2]. Viral hepatitis and nonalcoholic steatohepatitis are the most common causes of cirrhosis and approximately 80% of cases develop to HCC [3]. Due to the recurrence of HCC the prognosis of HCC remains discouraging and the 5-year overall survival rate which is only 34 to 50% [4]. Despite the rapid development of advanced medical technology, there are still no useful curable strategies for HCC patients [5]. Byeno et al. [6] reported that based on long-term survival data, serum OPN and DKK1 levels in patients with liver cancer can be deemed as novel biomarkers that show prognostic useful for liver cancer. Other serum markers, such as alpha-fetoprotein (AFP) and alkaline phosphatase (ALP or AKP), are proverbially used in clinical, but they lack sufficient sensitivity and specificity [7]. Therefore, finding useful biomarkers is indispensable for diagnosis and treatment for HCC patients.

Post-transcriptional modifications are essential for tumorigenesis and development. Centromere protein M (*CENPM*; otherwise called *PANE1, CENP-M* and *C22orf18*), which encodes a kinetic protein, binds to spindle microtubules to regulate chromosomal separation during cell division [8]. Expression of the *PANE1* gene was found preferentially in immune cells involving tumor tissues and tumor derived cell lines and leukemias and lymphomas [9]. Brickner et al. [10] found highly expressed in B lineage chronic lymphocytic leukemia (B-CLL) cells and resting CD19 (+) B cells, may be a potential therapeutic target for B-CLL. Bierie et al. [9] also demonstrated that human CENPM transcript cRNA was detected only in vivo or in vitro in activated B cells and T cells. These studies suggested *CENPM* may play critical role in tumor immune response and may be deemed to therapeutic target for immunotherapy. However, the role of *CENPM* in HCC prognostic remains unclear. In our study, we evaluated the expression of *CENPM* in HCC based on data from an online database to further understand the biological pathway of *CENPM* related to the pathogenesis of HCC. In addition, we also analyzed the connection between *CENPM* expression and clinical features as well as the correlation of its expression with immune infiltration level in HCC comes an online tumor infiltrating immune cells analysis tool.

## Materials and methods

### Data collection

Information on RNA-sequencing data (424 tissues, workflow type: HTSeqCounts) and comparative clinical data (377 patients, data format: BCR XML) were identified and got from the level 3 (standardized FPKM) of the TCGA-HCC cohort. Use boxplots to imagine expression differences for discrete variables [11]. The clinical factors included gender, stage, age, grade, T-phase, M-phase, N-phase, survival status and number of days of survival. Data analysis were checked by R (version 3.5.3) and R Bioconductor software packages.

### GSEA enrichment

Gene Set Enrichment Analysis (GSEA) created a list of all gene permutations related to CENPM expression. The samples were then divided into a high *CENPM* group and a low *CENPM* group as training sets to distinguish potential functions and use GSEA to clarify significant survival differences. Genome replacement is performed multiple times with each exam. The degree of expression of *CENPM* was used as a phenotypic marker. Normalized enrichment scores (NES) and nominal *P*-values have been used to classify the pathways of enrichment in each phenotype.

### Immune infiltrates analysis

TIMER [12] is a comprehensive database for the systematic study of immune infiltration in various malignant tumor types. The abundance of immune infiltrates (CD8+ T cells, B cells, CD4+ T cells, macrophages, neutrophils, and dendritic cells) was evaluated by our statistical methods and has been estimated using pathology Methods evaluated it. The network also enables users to explore the clinical relevance of one or more tumor immune subpopulations and has the flexibility to correct multiple covariates in a multivariate Cox proportional hazard model. Meanwhile, we contrast the differential level of *CENPM* between tumors and normal on all TCGA tumors.

### UALCAN and c-BioPortal analysis

UALCAN [13] is a user-friendly intelligent network asset for analyzing, discovering cancer data and in-depth analysis of TCGA gene expression information. One of the highlights of the portal is that it allows users to found between biomarkers or computer approval of potential genes of interest, and to evaluate genes in different clinical subgroups (such as gender, age, race, tumor grade, etc.) expression. cBioPortal [14] is an online free asset that can visualize, analyze, and download large-scale cancer transcription datasets. The portal included 245 cancer studies. The tab biological interaction network of *CENPM* and its co-expressed genes was got, and neighboring genes with altered frequencies were contained.

### TargetScan analysis

TargetScan [15] is a web for predicting potential biological targets of miRNAs. TargetScanHuman deems that the match to human 3′UTR and its orthologs is estimate by a UCSC genome-wide adjustment. As an alternative, they are ranked according to their predicted conservative positioning possibilities. FunRich [16] is a tool designed to process varieties of gene/protein datasets, in spite of the organism, and used for functional enrichment analysis. We used Funrich tools for miRNA enrichment analysis, including analysis of biological pathways, biological processes (BP), cellular components (CC) and molecular functions (MF).

### Statistical analysis

Scatter plots and paired plots visualize the differences between normal and tumor samples. Use delete ways to handle disappeared data, and if any individual value is disappeared, the data will exclude the full sample. The relationship between clinical factors and *CENPM* was used by logistic regression, Wilcoxon rank sum test, and Kruskal test. Multivariate Cox analysis was used to assess the effect of *CENPM* expression on survival and other clinical factors (such as age, gender, stage, distant metastasis). Benjamini–Hochberg's means of converting *P* values to FDR.

## Results

### Patients’ characteristics

The TCGA database contains 377 patients. The clinical and pathological properties of these samples are shown in Table 1. The middle age at diagnosis in TCGA was 53 years old (range 16–90 years) and median finally contact for subjects was 28.0 months (range 0–122.5 months). Meanwhile, follow-up for subjects conformed 129 (34.2%) alive and 248 (65.8%) death patients. Our study cohort included 122 (32.4%) female and 255 (67.6%) male patients. Stage I was located in 175 patients (46.4%), stage II in 87 (23.1%), stage III in 86 (22.8%) and stage IV in 5 (1.3%). Tumor stage was found T1 in 185 patients (49.1%), T2 in 95 (25.2%), T3 in 81 (21.5%) and T4 in 13 (3.4%). Node stage contained N0 in 257 (68.2%) and N1 in 4 (1.1%), 4 of 377 (1.1%) cases had distant metastases. All the subjects were adenomas or adenocarcinomas.

**Table 1:** TCGA hepatic carcinoma patient characteristics

Clinical characteristics	Total (377)	%
Age at diagnosis (year)	53 (16–90)	
Futime (month)	28.0 (0–122.5)	
Gender		
Female	122	32.4
Male	255	67.6
Stage		
I	175	46.4
II	87	23.1
III	86	22.8
IV	5	1.3
NA	24	6.4
Grade		
G1	55	14.6
G2	180	47.7
G3	124	32.9
G4	13	3.4
NA	5	1.3
T-classification		
T1	185	49.1
T2	95	25.2
T3	81	21.5
T4	13	3.4
TX	1	0.3
NA	2	0.5
M-classification		
M0	272	72.1
M1	4	1.1
MX	102	27.1
N-classification		
N0	257	68.2
N1	4	1.1
NX	115	30.5
NA	1	0.3
Status		
Alive	129	34.2
Death	248	65.8

Data express as mean (min–max)

### CENPM expression and clinical factors

**Fig. 1:**
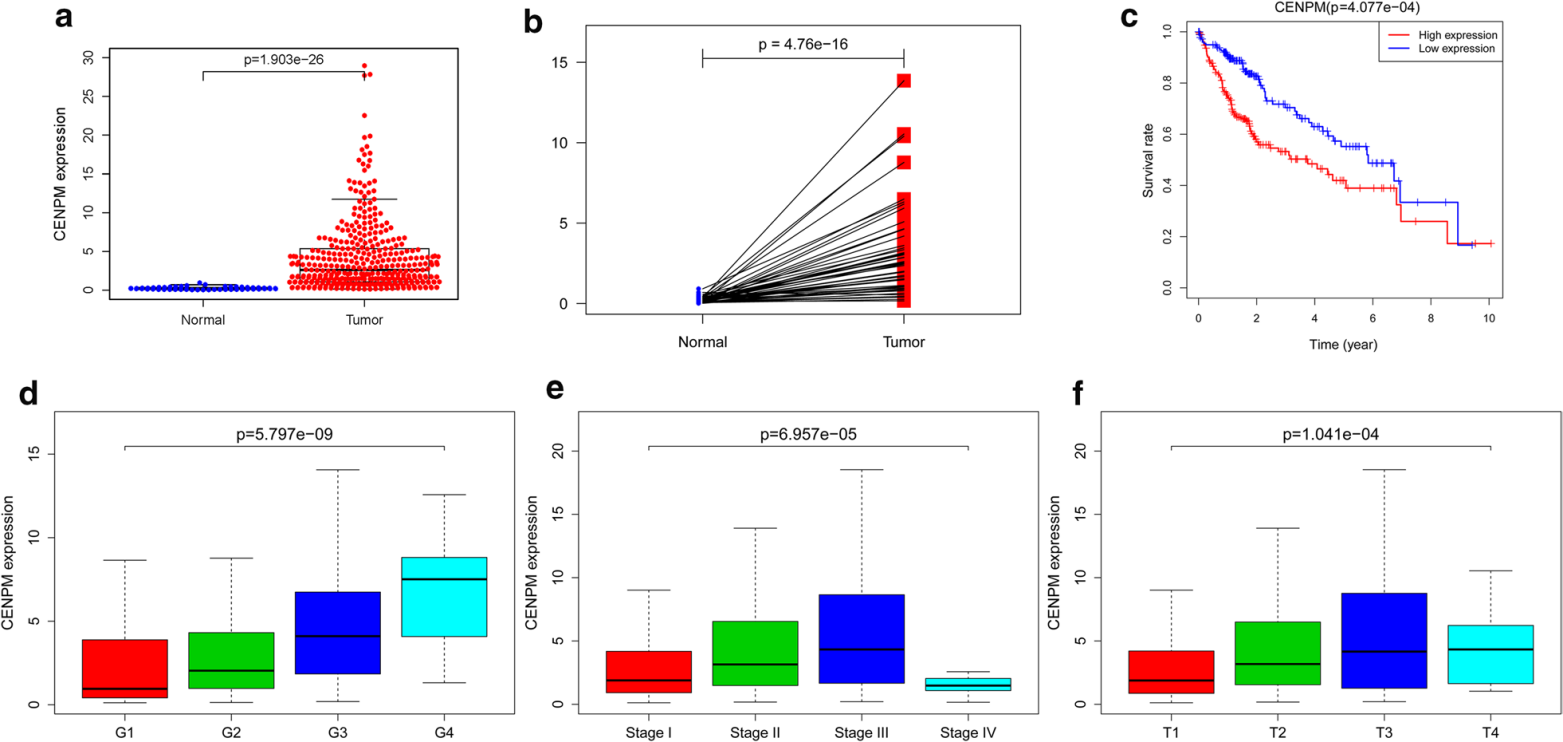
CENPM expression and the association among clinicopathologic factors. **a** The scatter plot showed the difference of CENPM expression between normal and tumor samples (P < 0.01); **b** paired plot to demonstrated the CENPM expression between normal and tumor from the same patients and the results was significant difference (P < 0.01); **c** Survival analysis. (P < 0.01); **d** Grade; **e** Stage; **f** T-stage

Scatter plot showing difference in *CENPM* expression among normal and tumor samples (*P* < 0.01), we then use paired plot to demonstrated the *CENPM* expression between normal and tumor from the same patients and the results was significant difference (*P* < 0.01) Fig. 1a, b. The outcomes suggested that the expression of *CENPM* was significant difference. The expression of *CENPM* correlated significantly with the patient grade (*P* < 0.01), clinical stage (*P* < 0.01) and T-classification (*P* < 0.01) Fig. 1d–f. Univariate analysis utilizing logistic regression uncovered that *CENPM* expression as a clear-cut ward variable was related to poor prognostic clinicopathologic factors (Table 2). CENPM expression in HCC as appreciably connected with grade (OR = 1.76; 95% CI 0.94–3.42, G1 vs. G3), stage (OR = 1.96; 95% CI 1.16–3.32, I vs. III) and T-classification (OR = 2.04; 95% CI 1.24–3.40, T1 vs. T3) indicated that patients with low *CENPM* expression are inclined to advance to a further advanced stage than those with high *CENPM* expression.

**Table 2:** CENPM expression associated with clinical pathological characteristics (logistic regression)

Clinical characteristics	Total (N)	Odds ratio in CENPM expression	P-value
Age (> 65 vs. ≤ 65)	376	1.20 (0.79–1.83)	0.390
Gender (female vs. male)	377	0.84 (0.54–1.29)	0.417
Grade (G1 vs. G3)	179	1.76 (0.94–3.42)	0.000
Stage (I vs. III)	260	1.96 (1.16–3.32)	0.000
T-stage (T1 vs. T3)	266	2.04 (1.24–3.40)	0.001

Categorical dependent variable, greater or less than the median expression level

### Survival and multivariate analysis

Survival analysis found that HCC with *CENPM*-high had a worse outcome than that with *CENPM*-low (*P* < 0.01) Fig. 1c. The univariate analysis suggested that *CENPM* linked essentially to stage (HR: 1.70; 95% CI 1.36–2.05; *P* < 0.01) and T-classification (HR: 1.64; 95% CI 1.35–2.01; *P* < 0.01) Table 3. Multivariate analysis showed that the expression of *CENPM* (HR = 1.03, *P* = 0.044) and M classification (HR = 1.38, *P* = 0.023) were independent prognostic factors for patients with HCC Table 3.

**Table 3:** a. Associations with overall survival and clinicopathologic characteristics in TCGA patients using Cox regression. b. Multivariate survival model after variable selection

Clinicopathologic variable	HR (95% CI)	P-value
a		
Age (continuous)	1.01 (1.00–1.03)	0.154
Gender (female vs. male)	0.81 (0.55–1.20)	0.298
Stage (I/II/III/IV)	1.70 (1.36–2.05)	0.000
Grade (G1/G2/G3/G4)	1.11 (0.86–1.44)	0.412
T-classification (T1/T2/T3/T4)	1.64 (1.35–2.01)	0.000
Distant metastasis (M0/M1/MX)	1.18 (0.95–1.47)	0.134
Lymph nodes (N0/N1/NX)	1.08 (0.86–1.34)	0.513
CENPM expression (high vs. low)	1.05 (1.02–1.08)	0.002
b		
Distant metastasis (M0/M1/MX)	1.38 (1.05–1.83)	0.023
CENPM expression (high vs. low)	1.03 (1.00–1.06)	0.044

### GSEA analysis

**Fig. 2:**
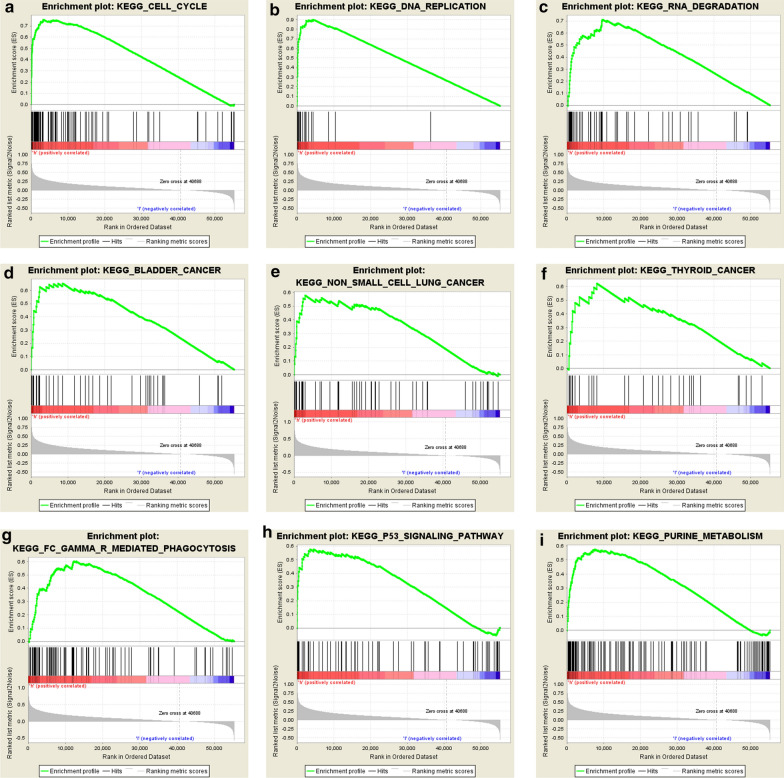
Enrichment plots from gene set enrichment analysis (GSEA)

To identify useful pathways that may be differentially initiated in liver cancer, we performed a GSEA analysis between low and high *CENPM* expression datasets. We chose the most abundant signaling pathway, depending on the standardized enrichment score (NES) Table 4. The results showed that *CENPM* high expression differentially enriched cell cycle, DNA replication, RNA degradation, certain cancers, phagocytosis, P53 signaling pathway and purine metabolism Fig. 2.

**Table 4:** Gene sets enriched in phenotype high

Gene set name	Size	NES	NOM P-val	FDR q-val
KEGG_CELL_CYCLE	124	2.13	0.000	0.002
KEGG_DNA_REPLICATION	36	2.08	0.000	0.002
KEGG_RNA_DEGRADATION	56	2.06	0.000	0.002
KEGG_BLADDER_CANCER	40	1.94	0.000	0.010
KEGG_NON_SMALL_CELL_LUNG_CANCER	54	1.78	0.008	0.029
KEGG_THYROID_CANCER	29	1.78	0.030	0.030
KEGG_FC_GAMMA_R_MEDIATED_PHAGOCYTOSIS	93	1.80	0.006	0.028
KEGG_P53_SIGNALING_PATHWAY	65	1.86	0.000	0.018
KEGG_PURINE_METABOLISM	152	2.10	0.000	0.002

NES: normalized enrichment score; NOM: nominal; FDR: false discovery rate. Gene sets with NOM P-val < 0.05 and FDR q-val < 0.25 are considered as significant

### Immune infiltrates related to CENPM in HCC

**Fig. 3:**
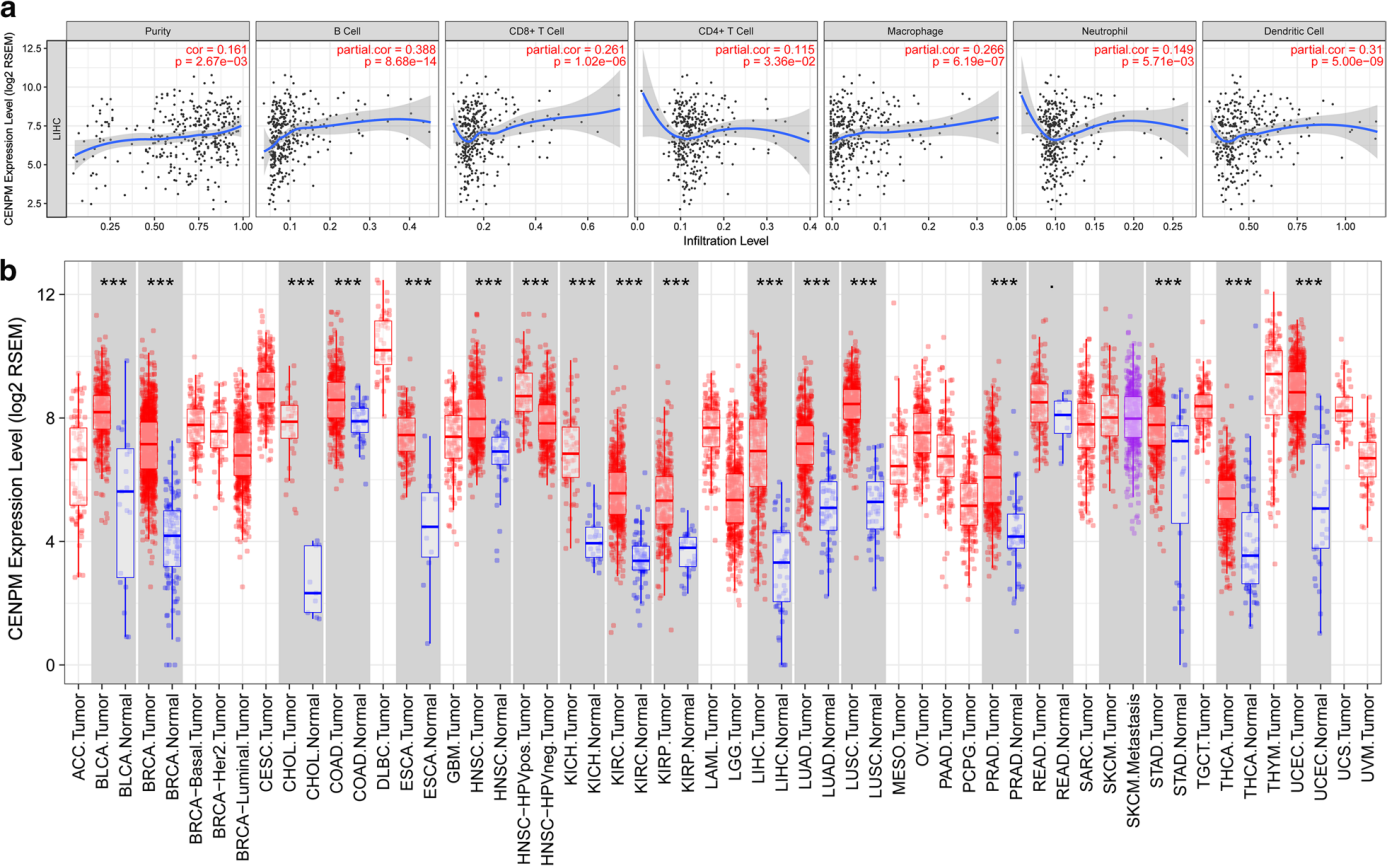
Immune infiltrates correlation with CENPM in HCC. **a** Correlation between CENPM in HCC expression and abundance of immune infiltrates (P < 0.05); **b** CENPM expression between various tumor and normal tissue

The correlation between *CENPM* in liver cancer expression and the abundance of immune infiltrates was statistically significant (*P* < 0.01, Fig. 3a). A multivariate Cox proportional hazard model showed that B-cells, CD8+ T cells, macrophages, and dendritic cells infiltrated by immune cells were statistically significant in liver cancer (*P* < 0.05), indicating that these immune cells significantly affect the prognosis, it is worth further research and exploration Table 5. At the same time, the expression of *CENPM* was also statistically significant (*P* < 0.05). Finally, we compared *CENPM* expression between various tumors and normal tissues. The results showed that *CENPM* was overexpressed in various cancers (*P* < 0.05, Fig. 3b).

**Table 5:** Multivariate survival model analysis based on TIMER online tool

Clinicopathologic variable	Coef	HR (95% CI)	P-value	Sig
Age	0.012	1.01 (1.00–1.03)	0.177	
Gender Male	− 0.071	0.93 (0.58–1.50)	0.769	
Race Black	1.185	3.27 (1.18–9.04)	0.022	*
Race White	− 0.032	0.97 (0.58–1.61)	0.902	
Stage II	0.174	1.19 (0.70–2.02)	0.522	
Stage III	0.711	2.04 (1.24–3.33)	0.005	**
Stage IV	1.434	4.19 (1.20–14.60)	0.024	*
Purity	0.561	1.75 (0.55–5.58)	0.343	
B cells	− 8.059	0.00 (0.00–0.59)	0.036	*
CD+ 8 cell	− 5.678	0.003 (0.00–0.50)	0.026	*
CD4+ T cells	− 6.886	0.001 (0.00–1.69)	0.069	·
Macrophages	8.002	2987.33 (11.72–761,175.73)	0.005	**
Neutrphils	− 1.906	0.15 (0.00–14,422.16)	0.745	
Dendritic	5.098	163.78 (3.78–7097.75)	0.008	**
CENPM	0.180	1.20 (1.04–1.38)	0.012	*

P-value significant codes: 0 ≤ *** < 0.001 ≤ ** < 0.01 ≤ * < 0.05 ≤ · < 0.1

### UALCAN and c-BioPortal analysis in HCC

**Fig. 4:**
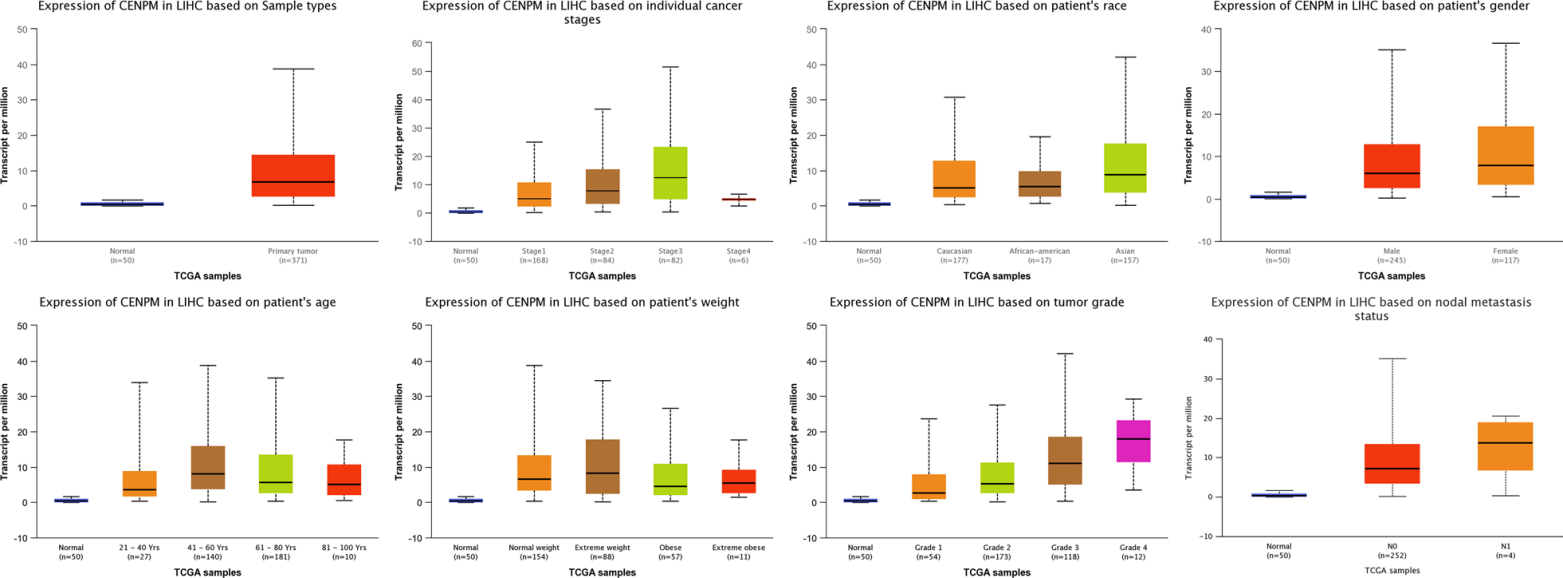
Boxplot showing relative expression of CENPM in subgroups of patients with HCC (UALCAN)

**Fig. 5:**
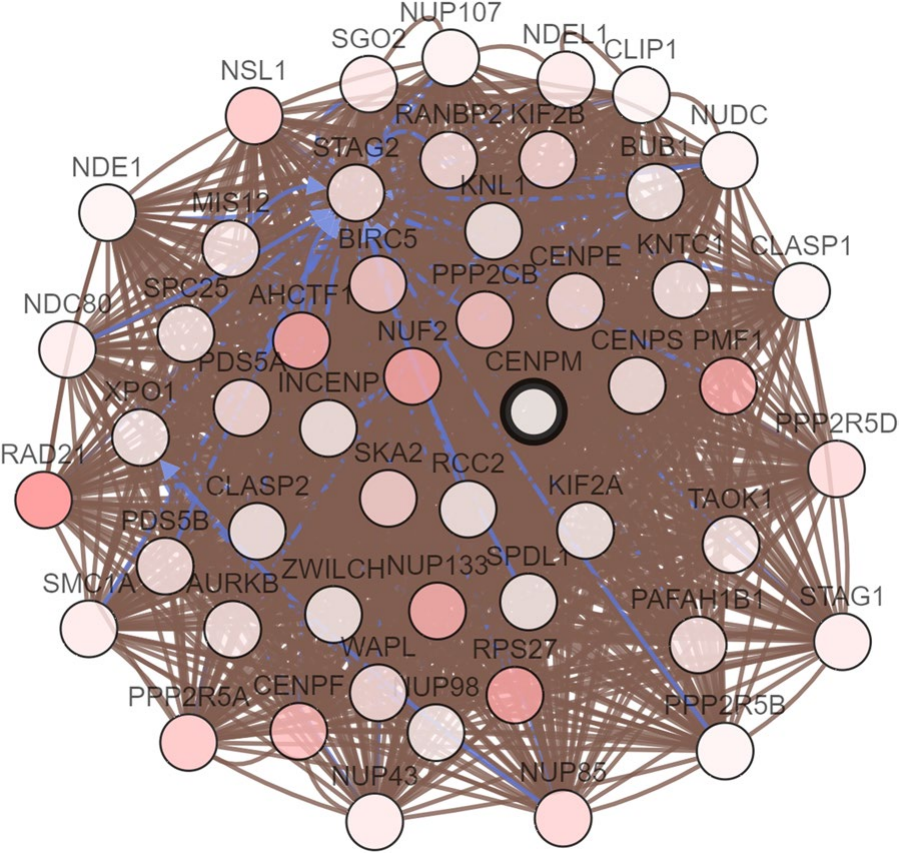
The network for CENPM and the 50 most frequently altered neighbor genes

In the age subgroup (normal age (21–40 years), normal age (41–60 years), normal age (61–80 years) and normal age (81–100 years)), among patients with liver cancer *CENPM* has substantially higher transcription levels than healthy individuals. Analysis in the weight subgroup; gender subgroup; ethnicity subgroup; tumor grade subgroups analysis also showed significantly higher *CENPM* in HCC patients (Fig. 4). In order to determine the biological interaction network of *CENPM* in liver cancer, we used the network in the "Network" tab in cBioPortal, showing the 50 most frequently changed neighbor genes in *CENPM*, and the most common change was *RAD21* (18.3%) **(**Fig. 5 and Table 6).

**Table 6:** The type and frequency of *CENPM* neighbor gene alterations in HCC (cBioPortal)

Gene symbol	Amplification	Homozygous deletion	Mutation	Total alteration
RAD21	17.8	0.3	0.3	18.3
RPS27	12.3	0.2	0.3	12.6
AHCTF1	9.6	0.3	2.2	12
NUF2	11.5	0.3	0.8	12.3
PMF1	12	0	0	12

### miRNAs related to CENPM

**Fig. 6:**
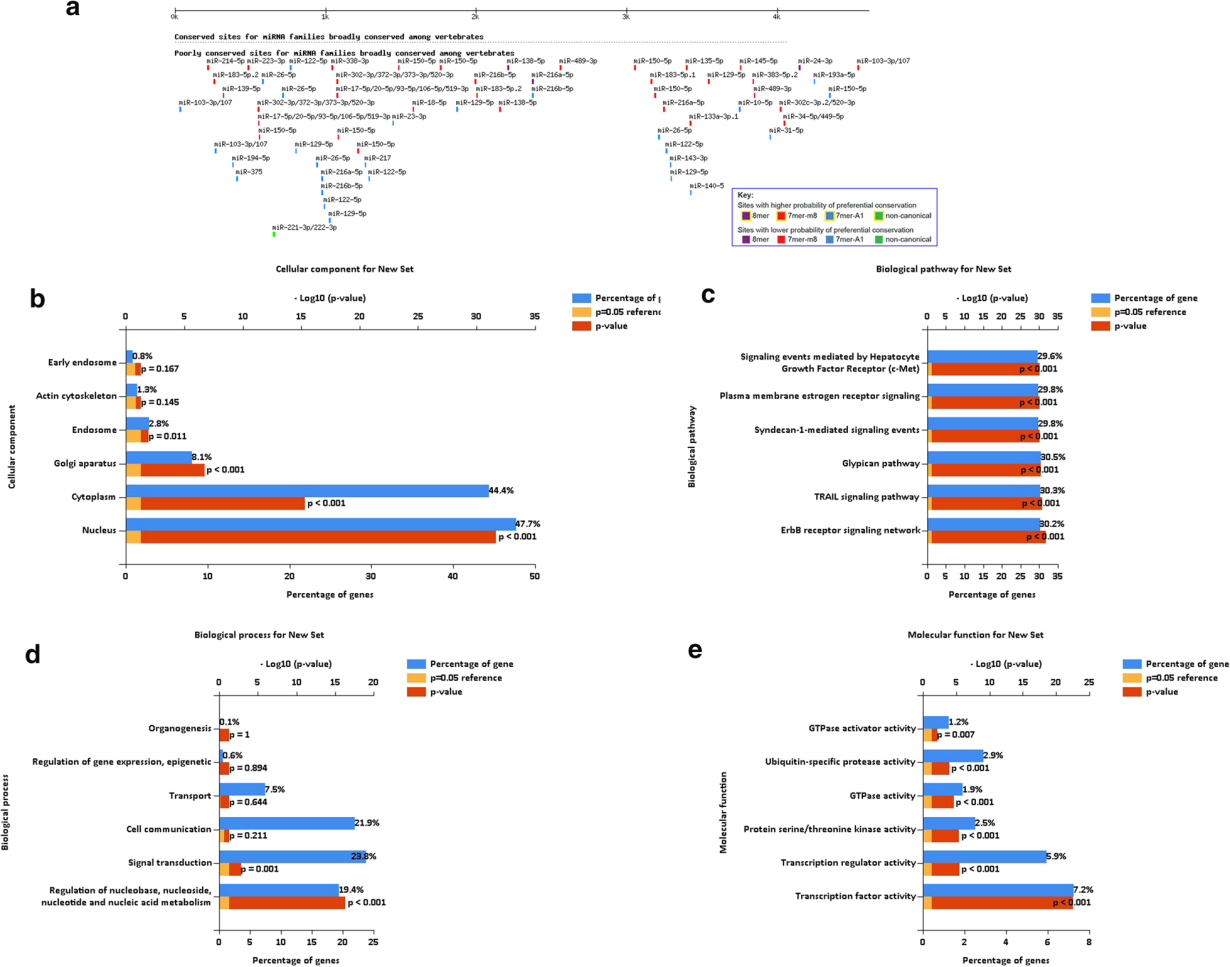
Enrichment analysis of the miRNA altered in the CENPM in HCC (Funrich and Targetscan). **a** Conserved sites for miRNA families broadly conserved among vertebrates. **b** Cellular components. **c** KEGG pathway analysis. **d** Biological processes. **e** Molecular functions

According to the online database, the top 3 of the 2081 miRNA families are hsa-miR-1307-5p, hsa-miR-449b-3p, and hsa-miR-6778-5p related to the gene *CENPM*. The conserved sites of the miRNA family that are widely conserved in vertebrates Fig. 6a. Using the Funrich database to explore the function of the identified 2081 miRNAs. BP are significantly enriched in the regulation of nucleobases, signal transduction, cell communication, transport, regulation of gene expression, and organogenesis. CC are mainly concentrated in the nucleus, cytoplasm, Golgi apparatus, endosome, actin cytoskeleton and early endosome. The MF are mainly transcription factor activity, transcription regulation activity, protein serine, GTPase activity and ubiquitin-specific protease activity, rich biological pathways in the ErbB receptor signaling network, TRAIL signaling pathway, Glypican pathway, and syndecan-1 mediated signaling events and signal transduction events mediated by hepatocyte growth factor receptor (c-Met) Fig. 6b–e.

## Discussion

In this work, we performed a detailed assessment of *CENPM* expression in hepatocellular carcinoma based on the TCGA database and explored its relationship with clinicopathological features, survival, function, immune infiltration, and expression differences. Understanding whether higher expression biomarkers in tumors are directly related to hepatocellular carcinoma can help us understand the mechanism of the observed clinical survival patterns. In our findings, the significant expression of *CENPM* suggests that *CENPM* may play an important role in regulating cancer progression. This should draw attention to current views on the improvement of liver cancer, and may reveal potential biomarkers or indicators to determine prognosis.

*CENPM* is an indispensable centromere protein involved in centromere assembly, which regulates mitochondrial protein assembly and chromosome segregation [17]. Huang et al. [18] cloned and identified the cDNA sequence of porcine *PANE1,* and found that porcine *PANE1* gene was expressed differently in seven different tissues, with the highest expression in lymph nodes and the lowest expression in kidney. Until now, the expression of *CENPM* and its potential prognostic effect on hepatocellular carcinoma has not yet been investigated, our outcomes showed that the expression of *CENPM* in hepatocellular carcinoma was related to advanced clinical pathologic factors (grade, clinical stage, T-classification), survival time, and poor prognosis. Univariate analysis uncovered that *CENPM* expression as a clear-cut ward variable was related to poor prognostic clinicopathologic factors and M-classification may play an indispensable role in the inclined to advance to a further advanced stage. The univariate and multivariate analysis also suggested *CENPM* still remained freely connected with OS and recommended that *CENPM* may act as a potential prognostic biomarker of prognosis and therapeutic target in hepatocellular carcinoma, but more researches needed to conduct for further study. In addition, we further analyzed various clinicopathological features of HCC samples using the UALCAN database, and all of them showed high transcription of CENPM.

To identify differential signaling pathways in liver cancer, GSEA analysis results show that cell cycle, DNA replication, RNA degradation, some cancers, phagocytosis, P53 signaling pathway and purine metabolism are differentially enriched in *CENPM* high expression phenotype. *CENPM* may influence cell cycle, DNA replication, RNA degradation then controls the begins and development of cancer cells. Kim et al. [19] was identified *CENPM* as a key gene that mediates the anti-cancer effect of garlic and cisplatin on bladder cancer, and showed that patients with low *CENPM* expressed better progression-free survival than patients without high expression. Studies also found the *CENPM* genes encode a human minor histocompatibility antigen expressed by tumor cells [9, 10]. Yu et al. [20] found *CENPM* could as AFP-related diagnostic biomarkers in HCC and validate the results using quantitative real-time PCR. Our study for the first time investigated the *CENPM* mRNA expression and its prognostic significance in hepatocellular carcinoma. Chen et al. [21] demonstrated that *LHX6* can inhibit the proliferation, invasion and migration P53 signaling pathways during hepatocarcinogenesis. Qin et al. [22] found that P53-stabilizing and activating RNA can strengthen the interaction between hnRNP K and P53, which ultimately leads to the accumulation and transactivation of P53. So *CENPM* may play a role via P53 signaling pathway and more researches needed to conduct in the future.

Previous studies demonstrated that human *CENPM* transcript cRNA was only detected in activated B- and T-cells either in vivo or in vitro. These studies suggested CENPM may play important role in tumor immune response so we used an online tool to analysis immune infiltrates correlation with *CENPM* in HCC. Multivariable Cox proportional hazard model showed that B cells, CD8+ T cells, macrophages and dendritic cells of immune infiltrates statistically significant (*P* < 0.05) in HCC indicating that these immune cells significantly affecting the prognosis. A latest study showed CD8+, CD68+, and FoxP3+ immune cells were associated with HCC, particularly in the invasive margin [23]. Macrophages not only promote the proliferation, colony formation and migration of HCC cells, but also maintain tumor growth and metastasis by secreting hepatocyte growth factor (HGF) [24]. Pang et al. [25] proposed that fusion of dendritic cells (DC) with tumor cells can effectively activate anti-tumor immunity in the body and affect tumor progression [26]. These studies indicate that *CENPM* may play an important role in tumor immune response and can be a good therapeutic target for immunotherapy.

To determine the biological interaction network of *CENPM* in liver cancer, we applied the 50 most frequently changed neighbor genes of CENPM on the Network tab in cBioPortal, and the most frequent change was *RAD21*. *RAD21* is a nuclear phospho-protein, which becomes hyperphosphorylated in cell cycle M phase. One study found that depletion of *RAD21* resulted in reduced levels of H3K27me3 at the Hoxa7 and Hoxa9 promoters, resulting in enhanced self-renewal of hematopoietic stem and progenitor cells (HSPC) [27]. Recent studies have shown that removing *RAD21* in a background lacking Pds5 can rescue the phenotype observed only in the absence of Pds5 [28]. Our study may provide information on adhesion kinetics in replication fork studies in patients with liver cancer. Our study also used the Targetscan online tool to distinguish *CENPM-*related miRNAs. To check the function of the identified miRNAs, bioenrichment was performed through the Funrich database. It is rich in ErbB receptor signaling network, TRAIL signaling pathway, Glypican pathway, syndecan-1 mediated signaling events and biological pathways of hepatocyte growth factor receptor (c-Met) signaling events. Studies have reported that selective c-Met inhibitors have antitumor activity in HCC and have acceptable safety and tolerability in Child–Pugh A liver function patients [29]. A recent study found that abnormal HGF/c-Met upregulation and activation are often observed in bladder cancer [30]. Studies have also found that metastasis associated with colon cancer 1 (*MACC1*) regulates *PDL1* expression and tumor immunity in gastric cancer (GC) cells through the c-Met/AKT/mTOR pathway [31]. We hypothesized that *CENPM* may regulate the expression of c-Met, leading to the occurrence of HCC, and more related research is needed. To date, this study demonstrates for the first time the important role of *CENPM* in the prognosis of hepatocellular carcinoma. However, future clinical trials are needed to validate these results and promote the use of *CENPM* in the prognostic evaluation of hepatocellular carcinoma.

## Conclusions

Our study found that the expression of *CENPM* was significantly increased in patients with hepatocellular carcinoma, and was related to a variety of clinical features, its correlation with the level of immune infiltration and poor prognosis, so *CENPM* may become a useful biomarker for the prognosis of patients with liver cancer.

## Data Availability

RNA-seq data and corresponding clinical data were acquired from the data portal for TCGA (https://portal.gdc.cancer.gov/).

## Abbreviations

HCC: Hepatocellular carcinoma
CENPM: Centromere protein M
GSEA: Gene set enrichment analysis
TCGA: Cancer genome atlas
GO: Gene ontology
KEGG: Kyoto encyclopedia of genes and genomes
BP: Biological processes
CC: Cellular components
MF: Molecular functions
OS: Over survival
